# Development of an enzyme immunoassay for detection of antibodies against *Coccidioides* in dogs and other mammalian species

**DOI:** 10.1371/journal.pone.0175081

**Published:** 2017-04-05

**Authors:** Nancy A. Chow, Mark D. Lindsley, Orion Z. McCotter, Dave Kangiser, Ron D. Wohrle, Wayne R. Clifford, Hayley D. Yaglom, Laura E. Adams, Kenneth Komatsu, Michelle M. Durkin, Rocky J. Baker, Lisa F. Shubitz, Gordana Derado, Tom M. Chiller, Anastasia P. Litvintseva

**Affiliations:** 1 Mycotic Diseases Branch, Centers for Disease Control and Prevention, Atlanta, Georgia, United States of America; 2 Zoonotic Disease Program, Office of Environmental Public Health Sciences, Washington State Department of Health, Olympia, Washington, United States of America; 3 Arizona Department of Health Services, Phoenix, Arizona, United States of America; 4 MiraVista Diagnostics, Indianapolis, Indiana, United States of America; 5 Oregon Veterinary Diagnostic Laboratory, Oregon State University, Corvallis, Oregon, United States of America; 6 Valley Fever Center for Excellence, University of Arizona, Tucson, Arizona, United States of America; 7 Biostatistics and Information Management Office, Division of Foodborne, Waterborne and Environmental Diseases, Centers for Disease Control and Prevention, Atlanta, Georgia, United States of America; University of Minnesota, UNITED STATES

## Abstract

*Coccidioides* is a soil-dwelling fungus that causes coccidioidomycosis, a disease also known as Valley fever, which affects humans and a variety of animal species. Recent findings of *Coccidioides* in new, unexpected areas of the United States have demonstrated the need for a better understanding of its geographic distribution. Large serological studies on animals could provide important information on the geographic distribution of this pathogen. To facilitate such studies, we used protein A/G, a recombinant protein that binds IgG antibodies from a variety of mammalian species, to develop an enzyme immunoassay (EIA) that detects IgG antibodies against *Coccidioides* in a highly sensitive and high-throughput manner. We showed the potential of this assay to be adapted to multiple animal species by testing a collection of serum and/or plasma samples from dogs, mice, and humans with or without confirmed coccidioidomycosis. We then evaluated the performance of the assay in dogs, using sera from dogs residing in a highly endemic area, and found seropositivity rates significantly higher than those in dogs of non-endemic areas. We further evaluated the specificity of the assay in dogs infected with other fungal pathogens known to cross-react with *Coccidioides*. Finally, we used the assay to perform a cross-sectional serosurvey investigating dogs from Washington, a state in which infection with *Coccidioides* has recently been documented. In summary, we have developed a *Coccidioides* EIA for the detection of antibodies in canines that is more sensitive and has higher throughput than currently available methods, and by testing this assay in mice and humans, we have shown a proof of principle of its adaptability for other animal species.

## Introduction

Coccidioidomycosis, also known as Valley fever, is a fungal disease caused by the soil-dwelling fungi *Coccidioides immitis* or *C*. *posadasii*. The disease has been characterized as endemic to the arid regions of southwestern United States, Mexico, Central America, and South America [1, 2]. Humans and a variety of mammalian species, including dogs, cats, horses, South American camelids, and marine mammals [3–7], can be infected when aerosolized arthroconidia (asexual spores) are inhaled. Although the precise ecological niche of *Coccidioides* remains unknown, it is widely accepted that the fungus grows in soil as hyphae and produces infective arthroconidia that become airborne upon soil disturbance [8–10].

Much of the work that mapped *Coccidioides* to the southwestern United States was derived from skin testing studies, which test for the presence of a delayed-type hypersensitivity response to *Coccidioides* indicating previous exposure. These studies were conducted in the 1940-50s on people who had no or limited reported travel outside of known endemic areas [11–14]. In addition, skin testing studies were performed on cattle as a sentinel species for human cases [15]. This approach was based on the assumption that animals travel considerably less than humans, and that exposure rates of a given area will reflect the presence of *Coccidioides* in that geographic area.

More recently, studies have used the gold-standard assay for antibody detection, agar gel immunodiffusion (AGID), to investigate dogs as a sentinel species for human cases [16]. Both prevalence and incidence of *Coccidioides* exposure in dogs were assessed in southern Arizona, an area known to be highly endemic for coccidioidomycosis [16]. In addition, dogs have been used in California and Texas to model the spatial distribution of *Coccidioides* and identify areas of high risk exposure for humans [17, 18].

Notably, recent findings demonstrating the presence of *Coccidioides* in the Pacific Northwest, specifically south central Washington, have challenged our current understanding of where this fungus resides and have highlighted the need to generate more accurate distribution maps [19–21]. Although surveillance methods that involve skin testing or serological assays that detect *Coccidioides* exposure in humans are available [11, 22, 23], it is becoming increasingly difficult to find people with limited travel history between known and unknown endemic areas. Testing animals susceptible to coccidioidomycosis may help to better understand the geographic distribution of this disease; however, few assays are available for testing animals. To date, there is no assay for detecting *Coccidioides* antibodies that is high-throughput, highly sensitive, and adaptable to a variety of animal species.

AGID is a highly specific test that can detect both IgM and IgG antibodies against *Coccidioides* in a variety of host species [22]; however, this method is time-consuming and is not suitable for large-scale surveillance studies. Conversely, EIAs are more sensitive, have a higher throughput, and can generate results in less than two hours compared to AGID, which requires 24–48 hours for incubation [24]. However, the main limitation of existing *Coccidioides* EIAs is that most of them are limited to detection in humans. The only reported study that used EIA for detection of anti-*Coccidioides* antibodies in animals other than humans is a report by Catalán-Dibene et al., who developed and field tested an EIA for testing rodents, using a mouse-specific secondary antibody. In addition, Durkin et al. developed a *Coccidioides* antigen EIA that can be applied to multiple host species [25], but a later investigation using this assay in dogs found antigen detection to be an insensitive method as compared to antibody detection [26].

In this study, we report the use of conjugated fusion protein A/G to develop an EIA that can detect antibodies against *Coccidioides* in a variety of animal species that are susceptible to coccidioidomycosis. Protein A/G is a recombinant protein capable of binding IgG antibodies from many animal species including dogs, cats, mice, rats, horses, alpacas, and rabbits. Previous studies have shown its multi-species use in immunoassays for detection of *Toxoplasma gondii* and *Borrelia* spp. [27–29]. For this assay, we demonstrated adaptability to dogs by testing serum samples from dogs with coccidioidomycosis and other endemic fungal infections, as well as from dogs residing in known endemic and non-endemic areas. We also performed a cross-sectional serosurvey investigating dogs in Washington, an area where burden of disease remains unclear and prevalence of *Coccidioides* exposure in dogs or any other animal species has not been investigated.

## Materials and methods

### Human and animal sera

All sera and/or plasma for the development and evaluation of this assay were residual specimens acquired from existing collections. No animal or human specimens were collected specifically for this project. All sera was shipped to the Centers for Disease Control and Prevention (CDC) frozen and stored for varying times at room temperature (RT), 4°C, and -80°C.

#### Sera for assay development

Canine sera positive for coccidioidomycosis (n = 37), blastomycosis (n = 10), and histoplasmosis (n = 10) were residual sera from anonymous specimens received after routine reference clinical testing at MiraVista Diagnostics Laboratory (Indianapolis, IN, USA). Residual murine plasma from C57BL/6 mice vaccinated with the attenuated *Coccidioides* and the uninfected controls were acquired from an unrelated study by the University of Arizona. Negative control sera from mice and healthy dogs residing in non-endemic areas were purchased from BioreclamationIVT (Chestertown, MD, USA). Human sera were from known *Coccidioides* seropositive and seronegative specimens from CDC collected under CDC IRB protocol exemption #4013, and consisted of anonymized sera collected as part of CDC public health surveillance efforts.

#### Sera for assay evaluation

To evaluate assay performance, we used sera from dogs that resided in known endemic regions of southern Arizona, sera from dogs that resided in non-endemic regions (S1 Table), and sera from dogs who resided in the newly identified endemic region Washington. Remnant dog sera from Arizona (n = 212) were received from a previous study by the Rickettsial Zoonosis Branch, CDC, which were obtained in three counties (Cochise, Santa Cruz, and Yuma) located near the U.S.-Mexico border. Serum was collected by rabies clinics, animal control centers, and animal humane societies. Information collected on the dogs included, age group (<1, 1–5, 6+ years), size (small, <20; medium, 20–55; large, 56+ pounds), county of residence (Cochise, Yuma, Santa Cruz), and ownership status (stray/free-roaming or owned/relinquished by the owner).

Samples from non-endemic areas (n = 663) were donated by two centers, Kansas State University (KSU) Veterinary Diagnostic Laboratory (Manhattan, KS, USA) and North Carolina State University (NCSU) College of Veterinary Medicine (Raleigh, NC, USA), and represented 36 states (S1 Table), all thought to be non-endemic for coccidioidomycosis. Serum was submitted by local providers for rabies (KSU) or tick-borne diseases (NCSU) diagnostic testing and surveillance and found to be seronegative for both. For both studies, history of travel to endemic areas for *Coccidioides* was not collected.

From Washington, remnant serum from dogs (n = 1041) obtained during routine veterinary examination was received from 18 veterinary clinics across the state with 88.8% (924/1041) of samples collected from the eastern arid regions of Washington. Clinics were asked to provide available residual serum samples and a residential zip code associated with the dog. Samples were collected during both routine healthy check-ups and during examinations for a variety of medical conditions.

### Protein A/G *Coccidioides* antibody EIA

The new protein A/G *Coccidioides* antibody EIA for the detection of IgG antibodies employed the use of the antigen coated plates from the OMEGA *Coccidioides* Antibody Enzyme Immunoassay (IMMY; Norman, OK, USA). This product was chosen over other commercially available products because the kit provides two separate plates for detection of IgG antibody (CF antigen-coated plate) and IgM antibody (TP antigen-coated plate) as compared to other kits that combine CF and TP antigen into one plate for simultaneous detection of IgG and IgM antibodies. The sensitivity of the assay should be improved using antigen coated plates that detects predominately IgG, as protein A/G does not bind to IgM.

#### Assay procedure

The manufacturer’s assay procedure was followed with the following exceptions: only the CF antigen-coated 96-well plate was used for antibody detection and EIA kit conjugate was substituted with a recombinant peroxidase conjugated protein A/G (Thermo Fisher Scientific, Rockford, IL, USA) for antibody detection. To perform the assay, 100 μl of the kit positive control, calibrator control, and animal serum diluted with the kit specimen diluent was added to the respective microwells of the CF antigen-coated plate. After a 30-min incubation at RT, microwells underwent three washes with the kit’s wash buffer using an ELx50 Microplate Strip Washer (BioTek, Winooski, VT, USA). After three washes, excess wash buffer was removed by striking the plate multiple times on paper towels. Protein A/G was diluted with the kit specimen diluent, and added to the microwells and left to incubate for 30 min. Three washes were performed again followed by striking the plate multiple times on paper towels, and 100 μl of the kit TMB substrate was then added to the microwells. After a 10-min incubation, 100 μl of the kit stop solution was added to each microwell and the optical density of each well was read at 450 nm with Molecular Devices SpectraMax 250 Microplate Reader (GMI, Ramsey, MN, USA). EIA units were calculated by normalizing the OD value of each sample by the OD value of the kit calibrator control. Thus, a sample with an EIA unit equal to 1.00 had an OD value equal to that of the calibrator.

#### Determination of optimal dilutions for protein A/G and sera

To determine the optimal concentration of the conjugated protein A/G for use in the EIA, serial 2-fold dilutions of protein A/G (1:10,000 to 1:80,000) were assayed against serially diluted *Coccidioides* or *Histoplasma* reagent control anti-sera developed for the use in AGID. At all protein A/G dilutions between 1:10,000 and 1:80,000, the results of the EIA assay were able to distinguish *Coccidioides* from *Histoplasma* when the AGID control anti-sera was diluted at 1:100 (S2 Table). The largest difference in EIA units was observed when using protein A/G at a dilution of 1:10,000; therefore, we chose this dilution for the remainder of the study. Next, we found the optimal serum dilution for this assay in dogs and mice to be 1:25 (S3 Table).

### Agar Gel Immunodiffusion (AGID)

AGIDs for detection of IgG antibodies against *Coccidioides* immunodiffusion complement fixation (IDCF) antigen was performed according to IMMY’s protocol using the *Coccidioides* Immunodiffusion reagents from IMMY. Specifically, *Coccidioides* IDCF Antigen (IMMY) and *Coccidioides* IDCF Positive Control (IMMY) were plated on precast Cleargel agar plates (IMMY). Plates were incubated in a humidified chamber at RT, and results were assessed in 72 hours.

### Data analysis

ROC curve analysis (performed using SigmaPlot 11.2, Sysat Software, Inc., San Jose, CA, USA) was used to determine the cutoff for the protein A/G EIA assay. Fisher exact chi-square test was used to compare proportions of dogs positive for IgG antibodies against *Coccidioides* from Arizona, Washington, and non-endemic areas. Simple logistic regression was used to compare the odds of testing positive for Cocci between geographic regions. Two sample *t*-test was used to compare mean EIA values between infected and healthy dog and mouse samples. Wilcoxon rank-sum test was used to compare median EIA values for human isolates, due to small sample size for human data. Exact McNemar’s test was used to compare the difference in dependent proportions from EIA and AGID tests. Univariable and multivariable logistic regression analyses were used to investigate the risk factors related to seropositivity. Statistical analyses were conducted using (SAS v. 9.4, SAS Institute, Cary, NC, USA). All p-values correspond to 2-sided tests, unless otherwise stated, and p-values of 0.05 or less were considered to indicate statistical significance.

## Results

### Use of protein A/G allows for detection of IgG antibodies against *Coccidioides* in multiple species

**Fig 1:**
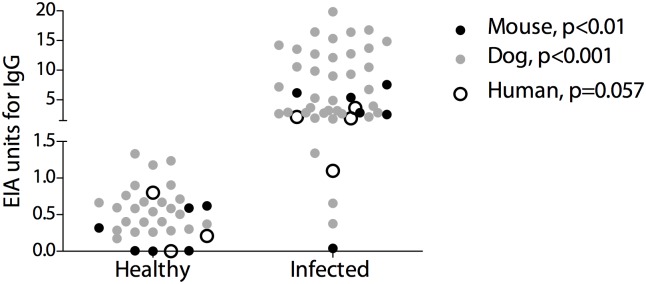
Detection of IgG antibodies against *Coccidioides* from multiple mammalian species using protein A/G. EIA units from healthy and *Coccidiodes*-infected mouse, dog, and human sera/plasma samples. The y-axis is split with a scale of 0–1.5 EIA units and of 1.5–20 EIA units.

We first assessed whether the protein A/G *Coccidioides* antibody EIA was able to detect IgG antibodies against *Coccidioides* from multiple mammalian species. Fig 1 demonstrates the assay’s ability to distinguish between healthy and *Coccidioides*-infected mice (p < 0.01), dogs (p < 0.001), and humans (p = 0.057).

### Development of the protein A/G EIA for *Coccidioides* using sera from naturally infected dogs

**Fig 2:**
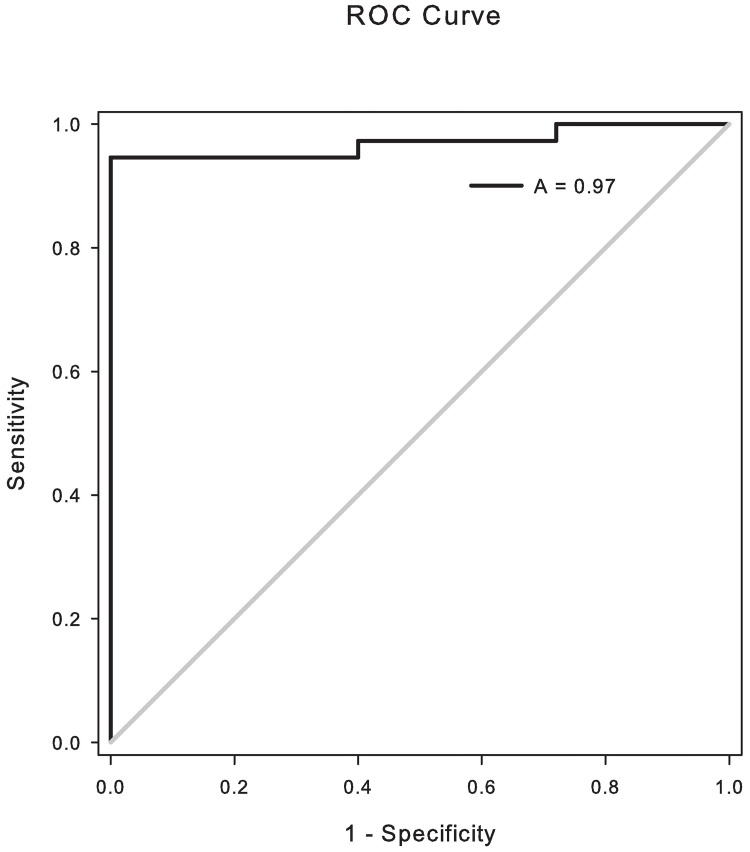
ROC curve analysis shows a high degree of diagnostic accuracy for the assay. Sensitivity and 1-Specificity were plotted for healthy dogs (n = 25) and dogs confirmed with coccidioidomycosis (n = 37). Estimated area under the curve (A) is 0.97.

The cutoff value of the protein A/G *Coccidioides* antibody EIA was determined by Receiver Operating Characteristic (ROC) curve analysis, a method that graphs sensitivity (true positive frequency) versus 1 –specificity (false positive frequency) across varying cutoffs in the unit square. Serum obtained from healthy dogs residing in non-endemic areas (n = 25) and from dogs with a confirmed diagnosis of coccidioidomycosis (n = 37) was analyzed. The estimated area under the ROC curve was found to be 0.97 (95% confidence interval (CI) = [0.93, 1.01], standard error = 0.02, p<0.001), indicating a high degree of diagnostic accuracy for the protein A/G *Coccidioides* antibody EIA (Fig 2). The cutoff value of 1.33 EIA units yielded a high degree of sensitivity and specificity for the assay and thus was used as the cutoff for the rest of our analysis.

At this cutoff value, 24 of 25 serum samples from healthy dogs tested negative for IgG antibodies against *Coccidioides*, for a specificity of 96.0% (95% CI = [86.3, 100], Table 1). Of the serum samples from dogs diagnosed with coccidioidomycosis, 35 of 37 tested positive for a sensitivity of 94.6% (95% CI = [81.8, 99.3], Table 1). Samples were also analyzed by AGID and compared to the protein A/G EIA (Table 2); agreement between the two assays was found to be 90.0% (56/62). By AGID, 30 of 37 serum samples from dogs diagnosed with coccidioidoymcosis tested positive for a sensitivity of 81.1% (95% CI = [64.8, 92.0]). All 25 serum samples from healthy dogs tested negative by AGID for a specificity of 100% (95% CI = [86.3, 100]). Comparing the EIA and AGID assays by an exact McNemar’s test resulted in a p-value of 0.014, thereby concluding that the tests are significantly different.

**Table 1:** Corresponding table for ROC curve analysis.

EIA diagnosis	Confirmed diagnosis		Cutoff	Area under the curve	Sensitivity % (95% CI^a^)	Specificity % (95% CI)
	Positive	Negative				
**Positive**	35	1	1.33	0.97	94.6 (81.8, 99.3)	96.0 (86.3, 100)
**Negative**	2	24				

^a^ Confidence Interval

**Table 2:** Comparison of AGID and EIA results for dog sera from confirmed coccidioidomycosis cases and healthy controls.

**Cases (n = 37)**	**AGID result**	**EIA result**	**EIA units**
**1**	+	+	19.84
**2**	+	+	16.77
**3**	+	+	16.44
**4**	+	+	16.40
**5**	+	+	15.34
**6**	+	+	14.85
**7**	+	+	14.20
**8**	+	+	13.72
**9**	+	+	13.55
**10**	-	+	12.76
**11**	+	+	12.71
**12**	+	+	12.11
**13**	+	+	10.56
**14**	+	+	10.49
**15**	+	+	9.85
**16**	+	+	9.31
**17**	+	+	9.02
**18**	+	+	7.18
**19**	+	+	6.75
**20**	+	+	5.30
**21**	+	+	4.89
**22**	+	+	3.95
**23**	+	+	3.68
**24**	+	+	3.21
**25**	-	+	3.17
**26**	+	+	2.90
**27**	+	+	2.84
**28**	+	+	2.82
**29**	-	+	2.77
**30**	-	+	2.68
**31**	+	+	2.68
**32**	+	+	2.12
**33**	+	+	1.91
**34**	-	+	1.79
**35**	+	+	1.34
**36**	-	-	0.65
**37**	-	-	0.38
**Controls (n = 25)**	**AGID result**	**EIA result**	**EIA units**
**1**	-	+	1.33
**2**	-	-	1.23
**3**	-	-	1.18
**4**	-	-	0.90
**5**	-	-	0.90
**6**	-	-	0.76
**7**	-	-	0.71
**8**	-	-	0.67
**9**	-	-	0.67
**10**	-	-	0.66
**11**	-	-	0.59
**12**	-	-	0.58
**13**	-	-	0.58
**14**	-	-	0.54
**15**	-	-	0.51
**16**	-	-	0.40
**17**	-	-	0.40
**18**	-	-	0.40
**19**	-	-	0.37
**20**	-	-	0.30
**21**	-	-	0.29
**22**	-	-	0.28
**23**	-	-	0.26
**24**	-	-	0.17
**25**	-	-	0.10

(+) and (-) represent positive and negative tests results, respectively.

To assess cross-reactivity of antibodies against other endemic fungal pathogens, we tested serum samples from dogs clinically diagnosed with two other diseases caused by dimorphic fungi (Table 3), histoplasmosis (n = 10) and blastomycosis (n = 10). For serum samples from dogs with confirmed histoplasmosis, 9 (90.0%) tested negative for IgG antibodies against *Coccidioides*. For serum samples from dogs with confirmed blastomycosis, all 10 tested negative.

**Table 3:** Sensitivity and specificity of EIA in dogs.

	Total	Positive	Negative
**Sensitivity**	37	35 (94.6%)	2 (5.4%)
**Specificity**	25	1 (4%)	24 (96%)
**Cross-Reactivity with *Histoplasma***	10	1 (10%)	9 (90%)
**Cross-Reactivity with *Blastomyces***	10	0 (0%)	10 (100%)

Positive and negative rates were assessed for healthy dogs (n = 25), dogs with confirmed coccidioidomycosis (n = 37), blastomycosis (n = 10), and histoplasmosis (n = 10).

### Use of the protein A/G EIA for veterinary testing of known and newly identified endemic regions

Using the protein A/G EIA, we estimated the prevalence of anti-*Coccidioides* antibodies among dogs from two states, one with a known level of endemnicity (Arizona) and another with an unknown level (Washington), as well as dogs from non-endemic areas. Of dogs residing in Arizona, 43/212 (20.3%) tested positive for IgG antibodies against *Coccidioides* (Table 4). In contrast, 30/663 (4.5%) of control dogs from non-endemic regions tested positive (Table 4). Arizona dogs had a significantly higher odds of testing positive compared to dogs from non-endemic areas (OR = 5.37, 95% CI = [3.27, 8.82], p<0.001, Table 4). The proportion of Arizona dogs that had antibodies against *Coccidioides* was higher among free-roaming or stray dogs (24/68; 30.8%) than those that were owned or relinquished by the owner (19/111; 14.6%).

**Table 4:** Comparison of EIA reactivity in sera from dogs residing in endemic (Arizona), non-endemic, and newly endemic (Washington) areas.

	Positive	Negative	OR^a^ (95% CI^b^)
**Arizona (n = 212)**	43 (20.3%)	169 (79.7%)	5.37 (3.27, 8.82)
**Washington (n = 1041)**	30 (2.9%)	1011 (97.1%)	0.63 (0.37, 1.05)
**Non-endemic states (n = 663)**	30 (4.5%)	633 (95.5%)	Ref ^c^

^a^ Odds ratio

^b^ Confidence Interval

^c^ Reference

Dogs that were free-roaming or stray had increased odds of testing positive compared to dogs who were owned or relinquished by their owner (OR = 2.59, 95% CI = [1.19, 5.66], p-value < 0.001), (Table 5). On univariable analysis, the other potential risk factors examined: age group, size, and county of residence were not significantly associated with seropositivity in dogs (Table 5). Furthermore, there were no significant interactions between ownership status and these other characteristics. On multivariable logistic regression analysis, after adjusting for age, OR for positivity increases to 2.86 (95% CI = [1.38, 5.93], p-value < 0.001).

**Table 5:** Comparison of characteristics between Arizona dogs with positive and negative presence of IgG antibodies.

Characteristics	Positive N (%) Total n = 43 (100%)	Negative N (%) Total n = 168 (100%)	Unadjusted Odds Ratio (95% CI^a^)
**Age (years)**			
<1	8 (25.0)	24 (75.0)	Ref ^b^
1–5	28 (18.9)	120 (81.1)	0.70 (0.29–1.72)
≥6	7 (22.6)	24 (77.4)	0.88 (0.27–2.80)
**Ownership Status**^c^			
Owned/Relinquished by Owner	19 (14.6)	111 (85.4)	Ref
Stray/Free-Roaming	24 (30.8)	54 (69.2)	**2.59 (1.31–5.15)**
**Size**			
Small (<20 pounds)	10 (15.4)	55 (84.6)	Ref
Medium (20–55 pounds)	18 (22.8)	61 (77.2)	1.62 (0.69–3.82)
Large (56+ pounds)	15 (22.4)	52 (77.6)	1.59 (0.66–3.85)
**County**			
Cochise	6 (15.4)	33 (84.6)	Ref
Yuma	18 (25.7)	52 (74.3)	1.90 (0.69–5.29)
Santa Cruz	19 (18.6)	83 (81.4)	1.26 (0.46–3.43)

Characteristics of age (years), ownership status, size, and county were assessed in Arizona dogs.

Four observations were omitted from analysis due to missing data.

^a^ Confidence interval

^b^ Reference

^c^ 3 Dogs were missing Ownership Status

To determine seropositive rates of dogs in Washington, serum from dogs (n = 1041) collected from 18 veterinary clinics within the state of Washington were tested and 30/1041 (2.9%) tested positive for antibodies to *Coccidioides* (Table 4). While dogs tested in Arizona have significantly higher odds of being positive, compared to non-endemic states, dogs tested in Washington did not have a significantly higher odds of testing positive for antibodies to *Coccidioides* compared to dogs from non-endemic areas (OR = 0.63, 95% CI = [0.37, 1.05], Table 4).

## Discussion

Identifying areas of geographic risk for acquiring an infection with *Coccidioides* is essential for healthcare providers and public health professionals to promptly identify persons with Valley fever. However, the available maps of endemic regions were developed in the 1940-50s by performing skin testing on cattle and people who had not left their county of birth and are likely outdated [14]. Replicating such methods in today’s more mobile population would be challenging. Therefore, additional methods, such as targeted environmental sampling and regional veterinary surveillance, are needed to identify the current geographic range of *Coccidioides*.

In an effort to contribute to updating *Coccidioides* distribution maps, we developed and validated an EIA that is capable of detecting IgG antibodies against *Coccidioides* that is sensitive, high-throughput, and adaptable to testing multiple mammals, including dogs. We demonstrated that a cutoff value of 1.33 EIA units for testing dog sera provided a high degree of sensitivity and specificity that were comparable to the reported rates for the original OMEGA *Coccidioides* Antibody Enzyme Immunoassay (IMMY) [30]. The assay was also highly specific with minimal signal detected from healthy controls, and animals with confirmed *Blastomyces* and *Histoplasma* infections. Specifically, of 20 animals with known *Histoplasma* and *Blastomyces* infections tested with our assay, only one cross-reacted with *Coccidioides*. However, this was not unexpected as cross-reactivity between *Coccidioides*, *Histoplasma*, and *Blastomyces* antigens has been reported previously (32, 33).

Of the control dogs residing in non-endemic areas for *Coccidioides*, 4.5% tested positive. This low level may be a result of cross-reactivity with *Blastomyces* and *Histoplasma*, which are endemic to the areas from which non-endemic sera were obtained. Alternatively, since history of travel for these dogs was unknown, it is possible that some of the seropositive dogs traveled into endemic regions and were exposed to *Coccidioides*. Recording travel history may improve the sensitivity of future surveys.

From our investigation of dogs with antibodies against *Coccidioides* from known endemic areas of the United States, we observed that in the endemic area of southern Arizona, 20.3% of tested dogs possessed antibodies against *Coccidioides*, as compared to 4.5% of dogs from the non-endemic area. In a similar cross-sectional study of southern Arizona dogs (4–18 months old) using AGID for detection, Shubitz et al. found 8% (32/381) of tested dogs to be positive for antibodies against *Coccidioides* [16]. The higher positivity proportion of 20.3% in our study is likely attributed to the greater sensitivity of EIAs compared to AGID. Other studies investigating risk factors associated with *Coccidioides* infection in dogs, identified increased risk in dogs that were kept outside and/or roamed on large areas of land (Butkiewicz et al., 2005). Similar to those studies, we found that dogs that were free-roaming or strays had a higher positive rate than owned or relinquished dogs which also suggests that more prolonged or intense time spent outdoors may increase *Coccidioides* exposure. The agreement between results obtained from different investigations further validate utility of our test for dog surveillance.

We investigated seropositivity rates of dogs residing in Washington where locally acquired cases have recently been identified. We found the seropositivity rate to be comparable between Washington dogs and dogs residing in other, non-endemic areas, thereby concluding that the degree of *Coccidioides* exposure in Washington dogs is low. These results showed that although locally-acquired human cases of coccidioidomycosis have occurred in Washington, the regional background IgG seropositive rate of this convenience survey was lower than an established endemic area. Continued surveillance could help track whether the range of *Coccidioides* expands or becomes more prevalent in areas of Washington. Based on our results from the Arizona population and previous studies, future studies might target stray or free-roaming dogs in contrast to this serosurvey that predominately tested sera from owned dogs visiting veterinary clinics.

One limitation of the assay is that it only detects IgG antibodies against *Coccidioides* in that protein A/G does not bind to IgM antibodies. Given that IgM antibodies against *Coccidioides* are associated with the initial antibody response during early primary infection [22] while IgG antibodies develop afterwards and persist longer as IgM levels wane, surveillance projects using the assay developed here would miss early acute *Coccidioides* infections. However, for the purpose of our study, the detection of IgG is more informative compared to IgM, as IgG levels are more likely to correlate with the previous exposure. Similarly, Shubitz et al. looked at both IgG and IgM antibodies against *Coccidioides* by AGID in dogs residing in Arizona and concluded that the shorter window of host IgM production and lower concentration of circulating IgM antibodies make it difficult to detect early *Coccidioides* infections with serological assays [16].

Finally, this assay can be used as a tool in investigating and mapping the expansion of the endemic region for coccidioidomycosis. We have validated the protein A/G EIA for *Coccidioides* detection in dogs, and demonstrated a proof of principle of its adaptability for other animal species. In the future, cutoff values for other species will need to be determined so that this method may be adopted for large serological studies in other mammals whose IgG antibodies are recognized by protein A/G, such as horses, cattle, cats, and others.

## Supporting information

S1 Data Data for Fig 1. (XLSX)

S2 Data Data for Fig 2 and Table 1. (XLSX)

S3 Data Data for Table 3. (XLSX)

S4 Data Data for Table 4. (XLSX)

S5 Data Data for Table 5. (XLSX)

S1 Table Represented non-endemic states for *Coccidioides*. Number of serum samples received from each non-endemic state (N = 36).
(DOCX)

S2 Table Determination of the optimal concentration of peroxidase-conjugated protein A/G for use in the EIA assay. Optimal concentration of protein A/G with a serum dilution of 1:100 was found to be 1:10,000. ^a^ dilution of Protein A/G; ^b^ Sera from rabbit inoculated with *Coccidioides*; ^c^ Sera from rabbit inoculated with *Histoplasma*; ^d^ EIA units (positive cutoff 1.33); ^e^ None Detected.
(DOCX)

S3 Table Determination of the optimal dilution of dog and mouse sera for use in the EIA assay. EIA results for healthy and *Coccidioides* infected dog (A) and mouse (B) sera at varying dilutions, 1/25 yielded the largest difference in EIA unites between health and *Coccidioides* infected species. ^a^ None Detected.
(DOCX)
