# Supplementary material for: Development of an enzyme immunoassay for detection of antibodies against Coccidioides in dogs and other mammalian species
Source: PLoS One. 2017 Apr 5;12(4):e0175081. doi: 10.1371/journal.pone.0175081 (PMC5381914; doi:10.1371/journal.pone.0175081)
Supplement: S1 Table — Number of serum samples received from each non-endemic state (N = 36). (DOCX) [file pone.0175081.s006.docx]

**S1 Table. Represented non-endemic states for *Coccidioides*.** Number of serum samples received from each non-endemic state (N = 36).

| State | No. of samples |
| --- | --- |
| Alaska (AK) | 2 |
| Alabama (AL) | 6 |
| Arkansas (AR) | 4 |
| Connecticut (CT) | 9 |
| District of Columbia (DC) | 4 |
| Delaware (DE) | 4 |
| Florida (FL) | 96 |
| Georgia (GA) | 36 |
| Iowa (IA) | 5 |
| Illinois (IL) | 23 |
| Indiana (IN) | 12 |
| Kansas (KS) | 8 |
| Kentucky (KY) | 13 |
| Louisiana (LA) | 6 |
| Massachusetts (MA) | 9 |
| Maryland (MD) | 30 |
| Maine (ME) | 4 |
| Michigan (MI) | 18 |
| Minnesota (MN) | 14 |
| Missouri (MO) | 22 |
| Mississippi (MS) | 9 |
| North Carolina (NC) | 58 |
| Nebraska (NE) | 2 |
| New Hampshire (NH) | 8 |
| New Jersey (NJ) | 23 |
| New York (NY) | 48 |
| Ohio (OH) | 31 |
| Oklahoma (OK) | 11 |
| Pennsylvania (PA) | 22 |
| Rhode Island (RI) | 1 |
| South Carolina (SC) | 16 |
| Tennessee (TN) | 13 |
| Virginia (VA) | 79 |
| Vermont (VT) | 1 |
| Wisconsin (WI) | 14 |
| West Virginia (WV) | 2 |
