# Supplementary material for: Development of an enzyme immunoassay for detection of antibodies against Coccidioides in dogs and other mammalian species
Source: PLoS One. 2017 Apr 5;12(4):e0175081. doi: 10.1371/journal.pone.0175081 (PMC5381914; doi:10.1371/journal.pone.0175081)
Supplement: S2 Table — Optimal concentration of protein A/G with a serum dilution of 1:100 was found to be 1:10,000. a dilution of Protein A/G; b Sera from rabbit inoculated with Coccidioides; c Sera from rabbit inoculated with Histoplasma; d EIA units (positive cutoff 1.33); e None Detected. (DOCX) [file pone.0175081.s007.docx]

**S2 Table**

**S2 Table. Determination of the optimal concentration of peroxidase-conjugated protein A/G for use in the EIA assay.** Optimal concentration of protein A/G with a serum dilution of 1:100 was found to be 1:10,000. ^a^ dilution of Protein A/G_;_ ^b^ Sera from rabbit inoculated with Coccidioides; ^c^ Sera from rabbit inoculated with Histoplasma; ^d^ EIA units (positive cutoff 1.33); ^e^ None Detected

|  | A/G 1:10,000^a^ | |  |  | A/G 1:20,000 | |  | A/G 1:40,000 | |  | A/G 1:80,000 | | |
| --- | --- | --- | --- | --- | --- | --- | --- | --- | --- | --- | --- | --- | --- |
| Rabbit serum dilution | *Coccidioides^b^* | *Histoplasma^c^* |  |  | *Coccidioides* | *Histoplasma* |  | *Coccidioides* | *Histoplasma* |  | *Coccidioides* | *Histoplasma* |  |
| 1:100 | 2.67^d^ | 0.12 |  |  | 2.43 | 0.12 |  | 2.18 | 0.09 |  | 1.4 | 0.08 |  |
| 1:1,000 | 0.32 | ND^e^ |  |  | 0.38 | ND |  | 0.20 | ND |  | 0.18 | 0.01 |  |
| 1:10,000 | 0.07 | ND |  |  | 0.01 | ND |  | ND | ND |  | ND | ND |  |
