# Supplementary material for: Development of an enzyme immunoassay for detection of antibodies against Coccidioides in dogs and other mammalian species
Source: PLoS One. 2017 Apr 5;12(4):e0175081. doi: 10.1371/journal.pone.0175081 (PMC5381914; doi:10.1371/journal.pone.0175081)
Supplement: S3 Table — EIA results for healthy and Coccidioides infected dog (A) and mouse (B) sera at varying dilutions, 1/25 yielded the largest difference in EIA unites between health and Coccidioides infected species. a None Detected. (DOCX) [file pone.0175081.s008.docx]

**S3 Table**

**S3 Table. Determination of the optimal dilution of dog and mouse sera for use in the EIA assay.** EIA results for healthy and *Coccidioides* infected dog (A) and mouse (B) sera at varying dilutions, 1/25 yielded the largest difference in EIA unites between health and *Coccidioides* infected species. ^a^ None Detected

**A.**

|  | Dog Sera Dilution | | | |
| --- | --- | --- | --- | --- |
|  | (1/25) | (1/50) | (1/100) | (1/200) |
| Healthy dog 1 | 0.41 | 0.22 | 0.10 | 0.03 |
| dog 2 | 0.83 | 0.44 | 0.19 | 0.08 |
| Infected dog 1 | 2.34 | 1.27 | 0.68 | 0.32 |
| dog 2 | 2.12 | 1.27 | 0.69 | 0.35 |
| dog 3 | 1.71 | 0.92 | 0.45 | 0.21 |
| dog 4 | 12.11 | 8.28 | 5.68 | 3.71 |

**B.**

|  | Mouse Sera Dilution | | | |
| --- | --- | --- | --- | --- |
|  | (1/25) | (1/50) | (1/100) | (1/200) |
| Healthy mouse 1 | 0.32 | 0 | 0.01 | ND |
| mouse 2 | 0.01 | ND^a^ | ND | 0 |
| mouse 3 | 0 | ND | ND | ND |
| mouse 4 | 0.01 | 0.02 | ND | ND |
| Infected mouse 1 | 7.56 | 4.80 | 2.59 | 1.34 |
| mouse 2 | 2.52 | 1.33 | 0.63 | 0.27 |
| mouse 3 | 6.18 | 3.98 | 2.07 | 1.13 |
| mouse 4 | 5.39 | 3.34 | 1.78 | 0.84 |
| mouse 5 | 0.04 | ND | ND | ND |
| mouse 6 | 2.83 | 1.48 | 0.75 | 0.32 |
| mouse 7 | 0.02 | ND | ND | ND |
| mouse 8 | ND | ND | ND | ND |
| mouse 9 | 0.06 | ND | ND | ND |

^a^ None Detected
